# Editorial: Recent advances in keratinocyte carcinomas: From molecular mechanisms to clinical perspectives

**DOI:** 10.3389/fmed.2022.1078020

**Published:** 2022-11-03

**Authors:** Constantin Caruntu, Aristidis M. Tsatsakis, Mircea Tampa, Simona-Roxana Georgescu, Salvador Gonzalez

**Affiliations:** ^1^Department of Physiology, Carol Davila University of Medicine and Pharmacy, Bucharest, Romania; ^2^Department of Dermatology, Prof. N.C. Paulescu National Institute of Diabetes, Nutrition and Metabolic Diseases, Bucharest, Romania; ^3^Forensic Science Department, Medical School, University of Crete, Heraklion, Greece; ^4^Department of Dermatology, “Carol Davila” University of Medicine and Pharmacy, Bucharest, Romania; ^5^Department of Dermatology, “Victor Babes” Hospital of Infectious Diseases, Bucharest, Romania; ^6^Department of Medicine and Medical Specialties, Alcalá de Henares University, Madrid, Spain

**Keywords:** non-melanoma skin cancer, squamous cell carcinoma, basal cell carcinoma, carcinogenesis, prevention, diagnosis, treatment

Keratinocyte carcinomas (KCs), including basal cell carcinoma (BCC) and squamous cell carcinoma (SCC) are the most common forms of cancer worldwide, and their incidence is showing a rapidly increasing trend in the last years (1, 2). Moreover, they are still associated with significant morbidity and mortality, even if the progress in diagnosis and treatment of keratinocyte-derived tumors is substantial (3–7). Hence, KCs emerged as an important topic of scientific interest and recent advances have been achieved.

In their paper, Kavasi et al. have reviewed impactful information regarding the structural and functional modulations of the KCs microenvironment. Moreover, the impact of extracellular matrix remodeling on the pathogenesis of non-melanoma skin cancers (NMSCs) was also discussed. The alteration of collagen matrix in NMSCs and the role of proteoglycans as mediators of tumor progression in KCs were emphasized, as well as the regulatory functions of matrix metalloproteinases (MMPs) in the tumor microenvironment. Especially the involvement of MMPs in the immune and inflammatory mechanisms in NMSCs has attracted the researchers' attention (8–13). Another important aspect regards the immune cells infiltrating the tumor microenvironment in KCs. In both BCC and SCC have been emphasized specific patterns of tumor infiltrating lymphocytes carrying prognostic significance (14–19). Moreover, alterations in circulatory MMPs and immune and inflammatory related elements were identified in NMSCs and could be used as potential markers for invasiveness and tumor progression (10, 20, 21). Thus, more and more information suggests the possible development of new biomarkers for diagnosis and prognosis, and new therapeutic strategies based on that matrix-related molecules and associated immuno-inflammatory factors (22, 23).

The impact of tumor microenvironment in the anti-tumor response and its modulatory factors in BCC have been investigated in a very interesting study. Byers et al. assessed the prognostic value of tertiary lymphoid structures (TLS), which are ectopic, lymphoid formations developed in the peritumoral regions (24). BCCs with nodular component showed a prominent presence of TLS. Moreover, an increased anti-tumor response was associated with more mature TLS. Furthermore, peritumoral stromal changes which correlated with the number of tumor infiltrating lymphocytes (TIL) were also identified, highlighting the contribution of the fibrillary matrix morphology on tumor lymphocytic infiltration. Previously, the significance of TLS was emphasized in other types of skin cancer (25–28), and now, the study of Byers et al. shows the presence of TLS in BCCs and demonstrates their modulatory connections with peritumoral stroma.

Ungureanu et al. discussed the role of dermoscopy in the assessment of BCC. Non-invasive imaging techniques play a significant part in both diagnosis and evaluation of therapeutic response in BCC (29). Dermoscopy in particular has gained a special place in clinical practice and allows the investigation of various parameters, thus increasing the specificity and sensitivity of the diagnosis (30–33). Various studies have also demonstrated the utility of dermoscopy for predicting the histological subtype of BCC, with an increased accuracy especially when used along *in vivo* reflectance confocal microscopy (34–37). Thus, dermoscopy can be employed as a reliable tool for choosing the optimal therapeutic approach in BCC. Moreover, it allows a more accurate evaluation of excision margins and, if a non-ablative treatment method was implemented, dermoscopy can be used for the assessment of the therapeutic response (38–41).

The risk factors involved in the development and progression of KCs and their mechanisms of action are other topics of interest (42). Ultraviolet radiation (UV) exposure is the most important environmental risk factor for skin carcinogenesis, and one of its mechanisms of action could be immunosupression, which is another recognized risk factor for KCs (43–49). Rollison et al. have investigated the association between Treg levels, UV exposure and the occurrence of skin SCC. Their results indicate a higher risk of skin SCC associated with increased levels of circulating CCR4^hi^ Tregs. The risk is further amplified if UV exposure was also present. Moreover, the presence of CCR4^hi^ Tregs in tumor tissue was correlated with markers of UV-induced damage such as solar elastosis.

The cellular and molecular mechanisms involved in KCs have been intensively investigated. Hsu et al. directed their attention toward a very complex subject: the impact of tripartite motif (TRIM) family proteins in cancer, with a special focus on the role of TRIM29 in cutaneous SCC and other related malignancies. Even if various studies have suggested a tumor-suppressive effect of TRIM29 in cutaneous and head and neck SCC (50–52), other evidence point out an association between TRIM29 expression and tumor development (53). Thus, further studies are needed in order to clarify the relationship between TRIM29 and tumorigenesis.

Actinic cheilitis (AC) is a common premalignant lesion associated with chronic UV exposure, primarily affecting the lower lip (54, 55). It has a broad spectrum of presentation and its transformation into invasive SCC can often be noticed too late. Thus, implementation of modern diagnostic tools allowing early detection and monitoring and new effective therapeutic strategies are essential to reduce the risk of malignant transformation and cancer progression and to increase the patients' quality of life (56–65). Vasilovici et al. have discussed the most important aspects regarding its epidemiological data, risk factors and clinical features. They also have presented recent findings on non-invasive imaging, diagnosis, and treatment options in AC.

Wei et al. have presented an interesting case of severe bilateral hyperkeratosis of the nipples and areolae, a rare skin disease characterized by a local warty thickening and pigmentation which must be differentiated from several malignancies such as mammary Paget disease of the skin or NMSCs (66). In addition, they have reviewed the most important information in scientific literature regarding this skin condition.

Thus, in this Research Topic, we brought together recent and relevant findings on KCs and related scientific fields. However, despite the considerable interest of the scientific community, there are still numerous gaps in understanding the complexity of KCs' pathogenesis. Therefore, we must increase our efforts to a better understanding of factors leading to the initiation and progression of KCs, to define new diagnostic methodologies, and to tailor more advanced personalized treatment protocols.

## Author contributions

All authors contributed to the manuscript drafting and revision. All authors read, and approved the submitted version.

## Conflict of interest

The authors declare that the research was conducted in the absence of any commercial or financial relationships that could be construed as a potential conflict of interest.

## Publisher's note

All claims expressed in this article are solely those of the authors and do not necessarily represent those of their affiliated organizations, or those of the publisher, the editors and the reviewers. Any product that may be evaluated in this article, or claim that may be made by its manufacturer, is not guaranteed or endorsed by the publisher.
